# Knowledge Based Cloud FE Simulation of Sheet Metal Forming Processes

**DOI:** 10.3791/53957

**Published:** 2016-12-13

**Authors:** Du Zhou, Xi Yuan, Haoxiang Gao, Ailing Wang, Jun Liu, Omer El Fakir, Denis J. Politis, Liliang Wang, Jianguo Lin

**Affiliations:** ^1^Department of Mechanical Engineering, Imperial College London

**Keywords:** Engineering, Issue 118, Knowledge Based Cloud FE (KBC-FE) simulation, sheet metal forming, hot stamping, high strength aluminum alloys, high temperature forming limit, coated tool life prediction

## Abstract

The use of Finite Element (FE) simulation software to adequately predict the outcome of sheet metal forming processes is crucial to enhancing the efficiency and lowering the development time of such processes, whilst reducing costs involved in trial-and-error prototyping. Recent focus on the substitution of steel components with aluminum alloy alternatives in the automotive and aerospace sectors has increased the need to simulate the forming behavior of such alloys for ever more complex component geometries. However these alloys, and in particular their high strength variants, exhibit limited formability at room temperature, and high temperature manufacturing technologies have been developed to form them. Consequently, advanced constitutive models are required to reflect the associated temperature and strain rate effects. Simulating such behavior is computationally very expensive using conventional FE simulation techniques.

This paper presents a novel Knowledge Based Cloud FE (KBC-FE) simulation technique that combines advanced material and friction models with conventional FE simulations in an efficient manner thus enhancing the capability of commercial simulation software packages. The application of these methods is demonstrated through two example case studies, namely: the prediction of a material's forming limit under hot stamping conditions, and the tool life prediction under multi-cycle loading conditions.

**Figure:**
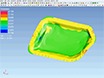


## Introduction

Finite Element (FE) simulations have become a powerful tool for optimizing process parameters in the metal forming industry. The reliability of FE simulation results is dependent on the accuracy of the material definition, input in the form of flow stress data or constitutive equations, and the assignment of the boundary conditions, such as the friction coefficient and the heat transfer coefficient. In the past few years, advanced FE simulations have been developed via the implementation of user-defined subroutines, which have significantly broadened the capability of FE software.

The use of such advanced FE simulations in the design of forming processes for structural components has been investigated by both the aviation and automotive industries, with the intention of producing lightweight structures that reduces operating costs and CO_2_ emissions. Particular focus has been placed on the replacement of steel components with lower density materials, such as aluminum alloys and magnesium alloys. However, these alloys, especially the stronger variants, offer limited formability at room temperature and thus complex-shaped components cannot be manufactured using the conventional cold stamping process. Therefore, advanced high temperature forming technologies, such as warm aluminum forming ^1-4^, hot stamping of aluminum alloys ^5-9^ and hot stamping of high strength steels ^10^, have been developed over the past decades to enable complex-shaped components to be formed. In general, high temperature forming processes involve significant temperature variations, strain rate and loading path changes ^11^, which would, for instance, cause inevitable viscoplastic and loading history dependent responses from the work piece materials. These are intrinsic features of high temperature forming processes and may be difficult to represent using conventional FE simulation techniques. Another desirable feature would be the ability to predict the tool life over multiple forming cycles in such processes, since they require low friction characteristics achieved through coatings that degrade with each forming operation. To represent all these features via the implementation of user-defined subroutines would be computationally very expensive. Moreover, the development and implementation of multiple subroutines would require excessive multi-disciplinary knowledge from an engineer conducting the simulations.

In the present work, a novel Knowledge Based Cloud FE (KBC-FE) simulation technique is proposed, based on the application of modules on a cloud computing environment, that enables an efficient and effective method of modeling advanced forming features in conjunction with conventional FE simulations. In this technique, data from the FE software is processed at each cloud module, and then imported back into the FE software in the relevant consistent format, for further processing and analysis. The development of these modules and their implementation in the KBC-FE is detailed.

## Protocol

### 1. Development of a High Temperature Forming Limit Prediction Model

1. Laser cut the specimens for formability tests from the aluminum alloy AA6082 sheets (1.5 mm thickness) into the selected geometries ^12^.
2. Etch a grid pattern, composed of 0.75 mm diameter circular points with a regular spacing of 1 mm, on the surface of the specimens using an electrolytic method ^13^.
3. Manually apply graphite grease as a lubricant on the non-etched side.
4. Assemble the dome test rig in a high rate hydraulic press ^12^. Use a 250 kN hydraulic universal testing machine.
5. Heat up the dome test rig to a testing temperature and set the punch at a constant moving speed. Then initiate the test. Note: The testing temperatures are 300, 400, and 450 °C, respectively. The testing speeds include 75, 250, and 400 mm/s.
6. Stop the test at the first occurrence of necking. Note: The press stroke (*i.e.,* final specimen height) is set such that necking is just observed on the formed specimen.
7. Measure the final specimen height using a height gauge, and calculate the strains and maximum strain rates (the rate of change of strain with respect to time) using an optical 3D forming analysis system. Analyze the changes in the grid spacing to compute the strains at each point of the formed specimen.
8. Ensure that the optical 3D forming analysis system includes a camera, the formed specimen, and calibration scale bars ^14^. Note: The specimen is placed at the center of a turntable and enclosed with the scale bars, and their relative positions are kept fixed for the duration of the analysis.
9. Set the camera at a fixed elevation (*e.g.,* 50 cm) and angle (*e.g.,* 30, 50, or 70°) to the specimen, and take pictures over a complete rotation (360°) of the turntable, in increments of 15°. Note: In the present work, three sets of images were acquired from multiple camera elevations and angles in order to map the strains over the entire specimen ^15^.
10. Load the images into the optical 3D forming analysis software, and proceed to compute the strains. Do this by clicking on the **'compute ellipses and bundle'** function, which detects the grid points, followed by clicking the **'compute 3D points and grid'** function which builds up the grid. Note: Calculate the strains and visualize it in the evaluation mode.
11. Output the strain distributions to determine the limit strains for each specimen based on ISO 12004 ^16^, and plot the forming limit diagrams for different forming speeds and forming temperatures.
12. Calibrate a material model for AA6082 at different temperatures from 300 to 500 °C and strain rates from 0.1 to 10 s^-1^. Note: The material model and its constants for AA6082 are detailed in reference ^17^.
13. Implement and unify the Hosford anisotropic yield function ^18^, Marciniak-Kuczynski (M-K) theory ^19^and the material model in step 1.12 into an integration algorithm so as to formulate the forming limit prediction model. Note: The model is described in reference ^11^.
14. Calibrate and verify the developed model for step 1.13 using the experimental results obtained in step 1.11.
15. Predict the forming limits through the verified model ^11^ from step 1.14. Note: **Figure 1** shows the resulting model predictions at different temperatures, at a forming speed of 250 mm/s, or equivalently, a strain rate of 6.26 s^-1^.

### 2. Development of an Interactive Friction/Wear Model

1. **Perform ball-on-disc tests for coated (disc) specimens**
  1. Prepare titanium nitride (TiN) coatings on bearing steel GCr15 disc using cathode arc and mid-frequency magnetron sputtering, with the deposition parameters given in reference ^20^.
  2. Using a scanning electron microscope (SEM), obtain surface/cross-section topography of the coated sample. Measure the TiN coating thickness through the SEM images by comparing the topography (brightness and contract) of base and coating materials. Note: The experimental procedures can be found in reference ^20^.
  3. Use a white light inter-ferometric surface pro-filometer to obtain the surface roughness of the sample. Place the sample under the lens and adjust the microscope to obtain clear surface structure. Illuminate the sample and adjust the angles of x and y axes to observe clear interference strips (which can be monitored from the screen). Set gross deepness in the software and start measurement. Automatically scan the sample surface and calculate the surface roughness.
  4. Evaluate the adherent strength of the sample using a micro-scratch tester. Apply an increasing load (maximum 50 N) and a scratch distance (maximum 5 mm) on the TiN coating. Determine the critical load causing failure of the coating and obtain the micro-scratch curves ^20^.
  5. Assess hardness of the sample using a hardness indenter. Apply a static load of 20 N on the sample for 15 s. Measure the diagonal of impression made by the indenter, and then obtain the hardness values from the tester.
  6. Conduct ball-on-disc tests on a tribometer in an ambient environment (temperature 25 °C, humidity 30%). Use a 6 mm diameter WC-6% ball (micro-hardness 1,780 HV, abrasion strength 1,380 N/cm, elastic modulus 71 GPa) as the counterpart against the coated disc. Adjust the relative sliding speed to 5 mm/s. Apply a normal load of 200 N. Start the motor and record friction values using the tribometer. Interrupt the test at 180 s, 350 s, 400 s, and 450 s, respectively, to analyze the wear track using an optical microscope ^20^.
  7. Measure the topography of the worn surface using a white light interferometric surface profilometer after testing.
  8. Repeat the tests (Step 2.1.6) with different normal loads (300 N, 400 N).
2. **Determine the evolution of the friction coefficient until the breakdown of the hard coating, characterized by a sharp increase in the friction coefficient**
  1. Plot the evolution of the friction coefficient against time after recording the friction values in Step 2.1.6. Note: The evolution of the friction coefficient is presented in reference ^20^.
  2. Assess the evolution of the friction coefficient in terms of wear behavior and the associated mechanisms. Note: The evolution of friction is characterized into three different stages: (i) low friction stage, (ii) ploughing friction stage, and (iii) coating breakdown stage ^20,21^.
  3. Evaluate the wear states at 180 s by manually interrupting the test, and then analyze the wear track using an optical microscope. Note: This step is to investigate the wear debris for the low friction stage as described in step 2.2.2.
  4. Repeat Step 2.2.3 at 350 s, 400 s, and 450 s, respectively.
3. **Develop the interactive friction model**
  1. Characterize the overall friction coefficient *µ* by combining the initial friction *µ*_α_ with the ploughing friction of hardware particles *µ*_Pc_ (as shown in Eq.(1)) ^20^. (1) 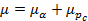  2. Combine the ploughing friction between the ball and substrate (*µ*_Ps_) with the instantaneous coating thickness (*h*) to model the coating breakdown induced sharp increase of the ploughing friction *µ*_Pc _(Eq.(2)). Note: In this case, *µ*_Pc _equals *µ*_Ps _when the remaining coating thickness is zero (indicating the complete breakdown of the hard coating). (2) 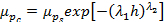 where*λ*_1_ and *λ*_2_ are model parameters introduced to represent the physical meaning of the wear process. *λ*_1_ describes the influence of large entrapped wear particles, and *λ*_2_ represents the intensity of the ploughing friction effect, which is characterized by the slope of the friction coefficient.
  3. Use a time based integration algorithm to obtain the evolution of the remaining coating thickness and model the accumulated wear under varying contact conditions. Update the coating thickness in each calculation loop by Eq. (3). (3) 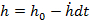 where *h*_0_ is the initial coating thickness and is the time dependent wear rate of the coating.
  4. Modify Archard's wear law ^22^ (Eq. (4)) and implement it in the present model. (4) 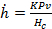 where *K* is the wear coefficient, *P* is the contact pressure, *v* is the sliding velocity, and *H*_c_ is the combined hardness of the coating and the substrate.
  5. Use Korsunsky's model to calculate the combined hardness (Eq. (5)). (5) 
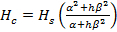
 where *H*_s_ is the hardness of the substrate, *α* is the hardness ratio between coating and substrate and β is the influence coefficient of the thickness.
  6. Represent the load dependent parameters *λ*_1 _and *K* by power law equations. (6) 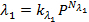 (7) 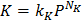 where *κ_λ1_*, *κ_K_*, *Ν_λ1_* and *Ν_K _*are material constants related to the evolution of friction ^20^.
  7. Fit the interactive friction model to the experimental results using an integration algorithm developed in the authors' group to determine the model parameters.

### 3. KBC-FE Simulation Case Studies

1. **KBC-FE simulation case study 1: prediction of forming limit under hot stamping conditions**
  1. Create and name a new simulation project in the FE simulation software. Select the process as '**Stamp hot forming**' and the solver type as '**PAM-AutoStamp**' when saving the project.
  2. Import the door inner die by clicking on the '**Import tools CAD**' and then '**Import & transfe**r' the door inner '**IGS**' geometry file into the FE simulation software graphic interface. Select the '**Hot forming**' strategy for meshing of tools. Name the imported object as '**Die**'.
  3. Repeat Step 3.1.2 and '**import**' the objects of Punch and Blankholder, respectively.
  4. Click on '**Blank**' under the '**Set-up**' tab. Click '**Add blank**' in the '**Blank editor**', and set the '**New object**' as '**Blank**'. Then select the type as '**Surface Blank**'.
  5. Choose '**Outline**' for the definition type and import the blank shape by clicking on '**Import from CAD file**'. Define '**Refinement**' as '**imposed level**' and select level 1 under '**Mesh options**'. Turn off '**Automatic meshing**' and set '**Mesh size**' to 4 mm.
  6. Define material properties in '**Blank editor**'. Click on '**Load a material**' under the '**Material**' tab. Select the '**AA6082**' (unit: mm·kg·ms·C) material as the material properties. Set the '**rolling direction**' to '**x = 1**'. Set the '**Blank thickness**' to 2 mm, and the blank '**Initial temperature**' to 490 °C. Note: The material properties and material model are described in reference ^17^.
  7. Click on '**Process**' under '**Set-up**' tab and select the '**+**' icon to load a new macro. Browse to '**\Stamp\Hotforming**' and select '**HF_Validation_DoubleAction_GPa.ksa**'. In the '**Customize**' dialog, activate the Blank, Die, Punch, and Blankholder. Under '**Stages**' tab, activate Gravity, Holding, Stamping, and Quenching.
  8. Set all parameters in the '**Objects attributes**' under '**Set-up**' tab to correspond with the actual experimental setup (blank holding force = 50 kN, forming speed = 250 mm/s, friction coefficient = 0.1, heat transfer coefficients ^23^ as a function of gap and contact pressure).
  9. Click '**Check**' icon to check the simulation set-up and ensure no errors in the above settings.
  10. Click '**Computation**' icon to start the simulation. Note: The software records 11 states during the simulation in a host computer.
  11. After completion of the simulation, observe the simulation results in the FE simulation software graphical interface, and proceed to record a '**script**' for an action exporting the contour values, *i.e.,* major strain (membrane), minor strain (membrane), and temperature of all the blank elements, for a specified simulation state. Click '**record**' and export contour values manually. Click '**stop**' to stop recording. Save the script so as to repeat the same action for all 11 simulation states.
  12. Click '**play**' icon to load the script, click '**Do All**' to export the contour values. Note: For each individual contour/state, the software automatically exports the values in '**ASCII**' files under '**major_strain_statenumber**', '**minor_strain_statenumber**', and '**temperature_statenumber**', respectively.
  13. Save all the exported files to a cloud computer. Run the '**necking prediction model**' (*i.e.,* cloud module code) together with all the exported files in the cloud computer.
  14. Predict the onset of necking through the use of forming limit prediction model in the cloud computer. Note: This model ^11^ gives users the option to run the prediction model on an individual element or all elements of the blank.
  15. Manually input the simulation details/parameters in the '**necking prediction model**'. Input the number of states in the simulation (state 11), total stroke of the stamping process (157 mm), stamping speed (250 mm/s), strain range of interest (the element selection criterion, *e.g.,* strain > 0.2) and all elements. Note: The strain range limits the elements for which necking may take place by setting an element criterion, *e.g.,* only the elements with a final major strain greater than 0.2 are selected for further evaluation in the module.
  16. After completing the module computation in the cloud computer, automatically save all the data (necking prediction results) into formatted '**ASCII**' files.
  17. Load the final state of the FE simulation results. Under the '**Contours**' tab, click on '**Imported**' and then '**Scalar values**'. Select the '**ASCII**' file obtained from the above step. Display the necking prediction results in the FE simulation software.
2. **KBC-FE simulation case study 2: tool life prediction under multi-cycle loading conditions**
  1. Create and name a new simulation project in the FE simulation software. Select the process as '**Standard stamping**' and the solver type as '**PAM-AutoStamp**' when saving the project.
  2. Import the die geometry by clicking on the '**Import tools CAD**' and then '**Import & transfer**' the U-shape die '**IGS**' geometry file into FE simulation software graphic interface. Select the '**Validation**' strategy for meshing of tools. Name the imported object as '**Die**'.
  3. Repeat Step 3.2.2 to import the objects of Punch and Blankholder, respectively.
  4. Click on '**Blank**' under '**Set-up**' tab. '**Add blank**' in the '**Blank editor**', set the '**New objec**t' as '**Blank**', and then select the type as '**Surface Blank**'. Choose '**Four points**' for the definition type and set the blank size to 120 × 80 mm^2^. Define '**Refinement**' as '**imposed level**': level 1 under '**Mesh options**'. Turn off  '**Automatic meshing**' and set '**Mesh size**' to 1.5 mm.
  5. Define material properties in '**Blank editor**'. Click on the '**Load a material'** under the '**Material**' tab. Select the '**AA5754-H111**' (unit: mm·kg·ms·C) material as the material properties. Set the '**rolling direction**' to '**x = 1**'. Set the '**Blank thickness**' to 1.5 mm.
  6. Click on '**Process**' under '**Set-up**' tab and select the '**+**' icon to load a new macro. Browse to '**\Stamp\Feasibility**' and select '**SingleActioin_GPa.ksa**'. In the '**Customize**' dialog, activate the Blank, Die, Punch, and Blankholder. Under '**Stages**', activate Gravity, Holding, and Stamping.
  7. Set all the '**parameters**' in the simulation to correspond with the actual experiment setup (blank holding forces = 5, 20, 50 kN, respectively, forming speed = 250 mm/s, friction coefficient = 0.17).
  8. '**Check**' the simulation set-up and ensure no errors in the above settings.
  9. Click on '**Computation**' icon and start the '**Computation**' for an 11-state U-shape bending simulation in a host computer.
  10. After completion of the simulation, export '**coordinate**' data and '**contact pressure**' data automatically for the work piece and tools (punch, die and blank holder) as '**ASCII**' files (as per Steps 3.1.11 and 3.1.12).
  11. Save all the exported files to a cloud computer. Run the '**tool life prediction module**' together with all the exported files in the cloud computer.
  12. Manually input forming parameters in the '**tool life prediction module**'. Input the following parameters: number of states (state 11), total stroke (70 mm), stamping speed (250 mm/sec) and initial friction coefficient (0.17).
  13. Select the tool (punch, die, or blank holder), and then start the computation for a single element or all the elements.
  14. After completion of the module computation in the cloud computer, automatically save all the data (including instantaneous remaining coating thickness and friction coefficient) into formatted '**ASCII**' files.
  15. Load and display the remaining coating thickness and friction coefficient for the relevant elements in the FE simulation software (as per Step 3.1.17).

## Representative Results

**KBC-FE Simulation for Necking Prediction**

In a hot stamping process, the use of a shape-optimized blank will not only save material cost but also help to reduce the presence of defects, such as necking, cracking, and wrinkling. The initial blank shape affects the material flow significantly during forming, and hence a sensible design of the blank shape is critical to the success of the hot stamping process and quality of the final products. To reduce the efforts of trial-and-error experiments to determine the optimal blank geometry, KBC-FE simulation was proven to be a highly efficient and effective method for minimizing the areas with necking. Using this technique, each simulation takes approximately 2 hours, while the parallel cloud module computation for necking prediction is completed within 4 hours.

**Figure 4** shows the evolution of the blank shape used in the hot stamping, an example of automotive door inner component. The initial blank shape, adopted from a conventional cold stamping process, was first used in the KBC-FE simulation. Experimental results in **Figure 4(a)** show that large failure (cracking or necking) areas are visible after the hot stamping. After one iteration of the blank shape optimization, it can be seen in **Figure 4(b)** that an almost fully successful panel is formed with much less necking, compared to using the initial blank shape. It can be seen that there is still an indication of necking at the pockets in the top right and left corners of the panel. After further optimization in **Figure 4(c)**, the optimized blank shape was finally obtained with no visible necking on the panel. The optimized blank shape determined by the KBC-FE simulation was verified experimentally through hot stamping trials conducted on a fully automated production line offered by a production system manufacturer.

**KBC-FE Simulation for Tool Life Prediction**

Conventional FE simulations of metal forming processes are performed for a single cycle. However, in a production environment, multiple forming cycles are performed on a given tool, where it is found that an increase in the number of forming cycles results in an increased variation between the formed components. This variation during multi-cycle tool loading is the result of changing surface topography. For example, the multi-cycle loading of forming tools with functional coatings will lead to a coating thickness reduction due to wear. Moreover, the breakdown of the coating will also be influenced by forming parameters, such as the load/pressure, forming speeds, *etc.* The KBC-FE technique enables the simulation of sheet metal forming processes under multi-cycle loading conditions, which is essential for the in-service life prediction of forming tools with advanced functional coatings.

To investigate the effects of blank holding force on the tool life, blank holding force values of 5, 20, and 50 kN were examined for a constant forming speed of 250 mm/s. **Figure 5** shows the remaining tool coating thickness distribution with different blank holding forces after 300 forming cycles. It clearly indicates that the remaining coating thickness decreases with an increase in the blank holding force.

**Figure 6** shows the pressure and remaining coating thickness distribution with blank holding forces of 5, 20, and 50 kN, respectively, along the curvilinear distance of the die after 300 forming cycles. Since the region A-B represents the die entrance region during the U-shape bending process, the pressure and the relative wear distance in this region were much higher than other regions of the die. Consequently, the wear of the coating mainly occurred in this area. There are two peak values of coating thickness reduction at 20 kN and 50 kN that correspond to the two peaks under the pressure. Meanwhile, the remaining coating thickness decreases with the increase of blank holding force. The lowest remaining coating thicknesses with blank holding forces of 5, 20, and 50 kN, were 0.905, 0.570, and 0.403 microns, respectively, where the initial coating thickness was 2.1 microns.


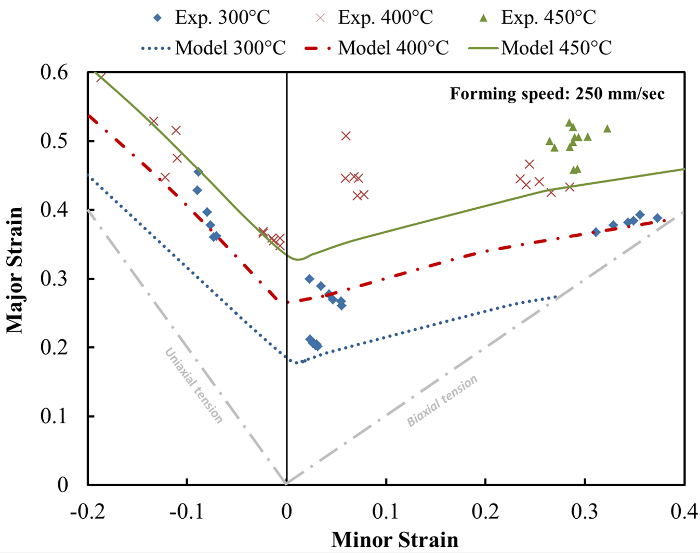
**Figure 1:Comparison between experimental and predicted forming limit strains at different temperatures.** The forming limit strains increase as temperature rises, at a constant speed of 250 mm/s, or equivalently, a strain rate of 6.26 s^-1^. Please click here to view a larger version of this figure.


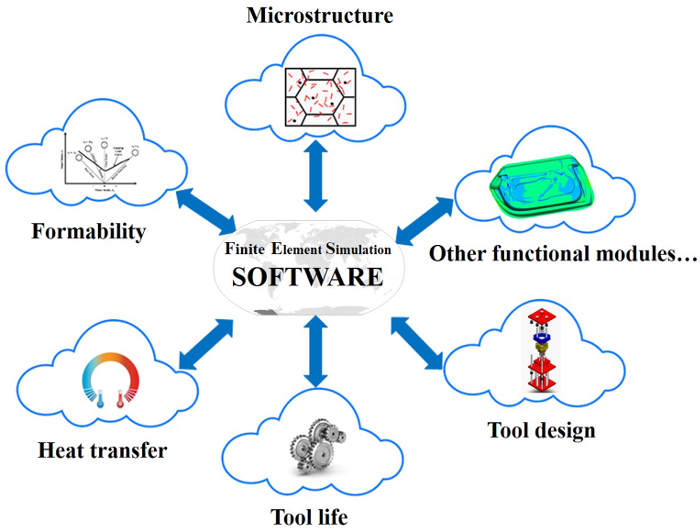
**Figure 2: Schematic chart for knowledge based cloud FE simulation of a sheet metal forming process. **Commercial FE simulation software, is used to run the simulation and export the results required for the individual modules. The modules, *e.g.*, formability, heat transfer, post-forming strength (microstructure), tool life prediction, tool design, *etc.*, work simultaneously and independently in the cloud, hence enabling the integration of cutting edge knowledge from multiple sources into FE simulations. Please click here to view a larger version of this figure.


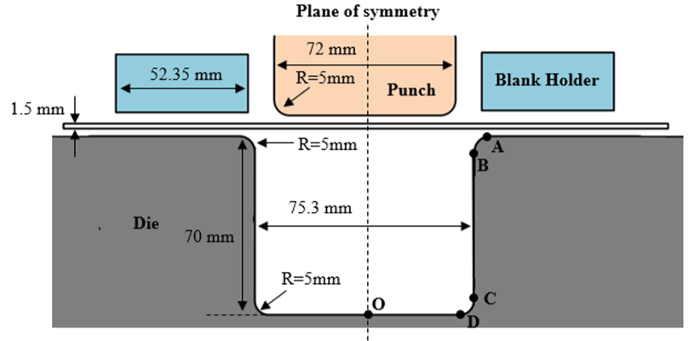
**Figure 3: Geometry of the work piece and tools for the U-shape bending simulation.** The tools, *i.e.,* punch, blank holder and die, are modeled using rigid elements. Shell elements are used for the work piece (blank) elements. Please click here to view a larger version of this figure.


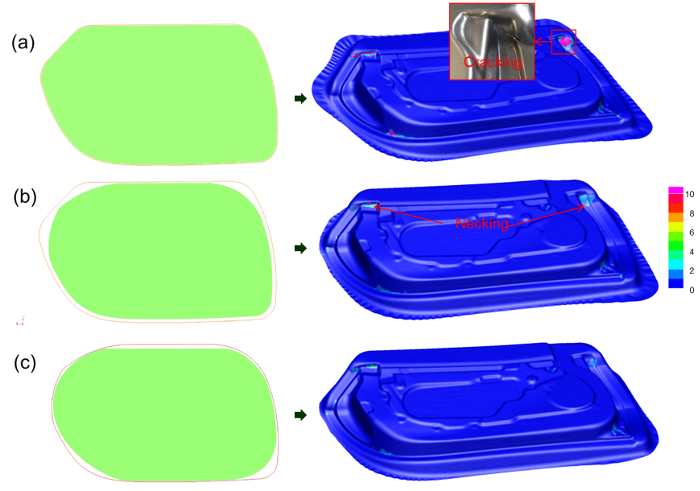
**Figure 4: Evolution of blank shape for hot stamping of a door inner panel (displayed in FE simulation). **Left:****The figures in green frames represent blank shapes at each optimization stage, and the ones in red frames correspond to the blank shape before its optimization. Right: Necking prediction results at each optimization stage. (**a**) Initial results with large failure (cracking/necking shown in red color), (**b**) Reduced failure with some necking after first stage of optimization, (**c**) Final optimized blank shape with no visible necking. Please click here to view a larger version of this figure.


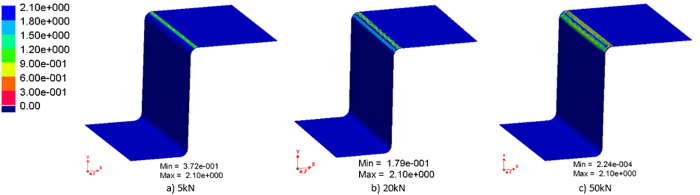
 **Figure 5: The remaining coating thickness distribution (displayed in FE simulation) with blank holding forces of: (a) 5 kN, (b) 20 kN, and (c) 50 kN, after 300 forming cycles at a constant stamping speed of 250 mm/s.** Please click here to view a larger version of this figure.


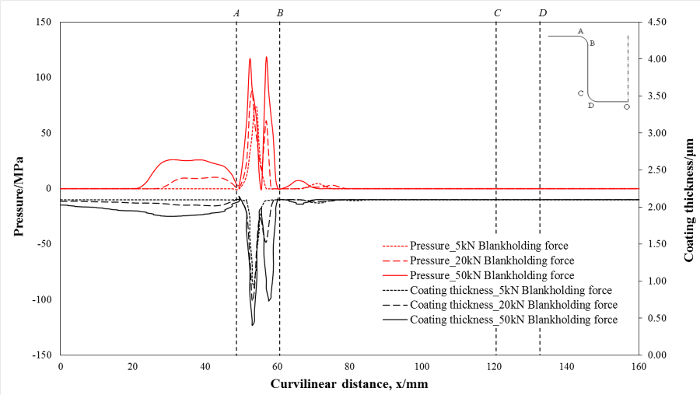
 **Figure 6: Prediction of contact pressure and remaining coating thickness with blank holding forces of: (a) 5 kN, (b) 20 kN, and (c) 50 kN, along the curvilinear distance of the die at a constant stamping speed of 250 mm/s.** Please click here to view a larger version of this figure.

## Discussion

The KBC-FE simulation technique enables advanced simulations to be conducted off site using dedicated modules. It can run functional modules on a cloud environment, that link up nodes from different specializations, to ensure that process simulations are conducted as accurately as possible. The critical aspects in the KBC-FE simulation may involve independency of the FE codes, efficiency of the computation, and accuracy of the functional modules. The realization of each advanced function in a module would rely on the development of a new model and/or a novel experimental technique. For example, the forming limit module is developed based on the new unified forming limit prediction model ^11^, and the friction tool life prediction module has currently been developed by the implementation of the interactive friction model ^20^. The KBC-FE simulation technique also offers the function of selective computation, *i.e.*, only the elements fulfilling the selection criteria are selected for further evaluation in the individual modules. For instance, the tool life prediction module automatically selects the elements for which the hard coating tends to breakdown, by ranking the wear rate of all the elements in the 1st forming cycle, thus usually less than 1% of the elements will be selected for further tool life evaluations under multi-cycle loading conditions. In the present research, the tool life prediction after 300 forming cycles can be completed within 5 min.

By conducting the relevant tests and calibrating accordingly, the forming limit model could be applied to forming process simulations to consequently determine the optimal parameters for producing a component from such alloys successfully, and with no incidences of necking. The forming limit prediction model was developed as a cloud module that was independent of the FE software being utilized, and could be applied to any FE software to assess the formability of a material during forming, without complicated subroutines ^17^. By importing the relevant data into the model, calculations could be carried out to determine whether failure would occur, in regions of the component that the user could specify, saving on computational resources. However, it should be noted that as the stress-strain curves are input into the FE software through a simple look-up table, it may be difficult to fully represent the material properties at various temperatures and strain rates during simulation. 

In the tool life prediction module, the frictional behavior during forming can be predicted by importing the required deformation history data into the verified friction module^ 20^, and then importing the discrete data points calculated by the cloud module for each element back into the FE software. This ensures that the advanced friction module can be used by all FE codes, regardless of their ability to incorporate user-subroutines. Additionally, the module could be run in parallel to further reduce the computation time. The interactive friction/wear model assumed the absence of wear particles during initial sliding, and as a result, it would be reasonable to expect a constant initial value of friction coefficient 0.17 ^20^. Although this model revealed the evolution of friction distribution, the frictional behavior during a forming process is very complicated, and it is difficult to completely integrate the complex frictional behavior from the cloud module into the FE simulation.

As a future technology, the KBC-FE simulation will rely on the development of dedicated and robust internet based FE simulation software packages, which would require a highly profitable, but completely different business model to be established by the software developers. In addition, a dedicated internal network needs to be built within the collaborative parties to ensure data security and the control reliability of the industrial system.

## Disclosures

The authors have nothing to disclose.
